# Altered Populations of Unconventional T Cell Lineages in Patients with Langerhans Cell Histiocytosis

**DOI:** 10.1038/s41598-018-34873-y

**Published:** 2018-11-07

**Authors:** Jenée Mitchell, Egle Kvedaraite, Tatiana von Bahr Greenwood, Jan-Inge Henter, Daniel G. Pellicci, Stuart P. Berzins, George Kannourakis

**Affiliations:** 1 Fiona Elsey Cancer Research Institute, Ballarat, Australia; 2 Federation University Australia, Ballarat, Australia; 3 Childhood Cancer Research Unit, Department of Women’s and Children’s Health, Karolinska Institutet, Stockholm, Sweden; 4 Karolinska University Hospital, Stockholm, Sweden; 5 Murdoch Children’s Research Institute, Parkville, Australia; 6 Department of Microbiology and Immunology, Peter Doherty Institute, University of Melbourne, Parkville, Australia

## Abstract

Langerhans cell histiocytosis (LCH) lesions are defined by the presence of CD1a^+^/CD207^+^ myeloid cells, but many other immune cells are present including unconventional T cells, which have powerful immunoregulatory functions. Unconventional T cell lineages include mucosal-associated invariant T (MAIT) cells, type I natural killer T (NKT) cells and gamma-delta (γδ) T cells, which are associated with many inflammatory conditions, although their importance has not been studied in LCH. We characterized their phenotype and function in blood and lesions from patients with LCH, and identified a deficiency in MAIT cell frequency and abnormalities in the subset distributions of γδ T cells and NKT cells. Such abnormalities are associated with immune dysregulation in other disease settings and are therefore potentially important in LCH. Our study is the first to recognize alterations to MAIT cell proportions in patients with LCH. This finding along with other abnormalities identified amongst unconventional T cells could potentially influence the onset and progression of LCH, thereby highlighting potential targets for new immune based therapies.

## Introduction

Langerhans cell histiocytosis (LCH) is a rare disease that most frequently affects children but can also occur in adults^1^. LCH is characterized by inflammatory lesions affecting one or more organs. Osseous and cutaneous tissue are the sites most frequently affected while liver, spleen and hematopoietic involvement are associated with an increased mortality risk^2^. All LCH lesions contain myeloid lineage cells that express CD1a and CD207^2–4^. These ‘LCH cells’ form the characteristic LCH microenvironment alongside a cellular infiltrate of T cells, macrophages, eosinophils, neutrophils, B cells, plasma cells and multinucleated giant cells^3,5^. Immune cells are fundamental to the inflammation and subsequent organ damage seen in LCH, but the role of different lineages is not well understood and they have not been specifically targeted in therapies. Recent advances in our understanding of immune regulation coupled with the development of new immunotherapies suggests that immune cells within LCH lesions may be potential targets for new treatments.

Patients with LCH often have mutations in the mitogen-activated protein kinase (MAPK) cell signalling pathway^6–12^ and show signs of immune dysregulation^5,13–15^, although the nature of these defects and their significance to the etiology of LCH is not fully understood. The composition and inflammatory characteristics of LCH lesions suggest a localised dysregulation of immune cells and a link between innate and adaptive immunity at the site of inflammation in LCH remains to be elucidated. The characteristic presence of LCH cells implies their involvement in the pathogenesis of this disease and it is possible that they promote T cell signalling that leads to potent cytokine release within lesions. This hypothesis is consistent with the large number of activated T cells within LCH lesions^5,13,16^.

Interestingly, there are also signs of immune abnormalities outside of the lesions of patients with LCH. For example, there are reports of CD1a^+^ LCH-like cells with myeloid characteristics^1,17,18^ and increased Foxp3^+^ regulatory T cells (Tregs)^14^ in the circulation of patients with active LCH that suggest altered immune regulation in LCH. Other lineages of immune cells with known regulatory functions have not been well-studied in LCH patients. Prime candidates to investigate are unconventional T cells such as mucosal associated invariant T (MAIT) cells, gamma delta (γδ) T cells and type I natural killer T cells (referred to herein as NKT cells), which are all capable of rapid inflammatory cytokine responses that can trigger and potentiate innate and adaptive immune responses. These unconventional T cells are already proposed to play fundamental roles in regulating aspects of tumor immunity, infection and autoimmunity^19–23^, piquing our curiosity about their role in LCH. One study found γδ T cells at a high frequency in LCH lesions^24^ and there is overexpression of *CD1D* in LCH cells compared with skin-resident Langerhans cells (LCs)^25^. NKT cells respond to lipids presented by the major histocompatibility complex (MHC)-like molecule, CD1d, hence higher expression of *CD1D* in lesions suggests a potential role for NKT cells in LCH. MAIT cells were first associated with anti-microbial responses, but have more recently been found to have an altered function in colorectal tumors^22,23^ and type 2 diabetes^26^, and they are associated with several autoimmune diseases^27,28^. Like NKT cells, MAIT cells have not been studied in LCH, but expression of CD161 is a defining characteristic of MAIT cells, and it is noteworthy that the gene encoding for the C-type lectin receptor for CD161 (*CLEC2D*) is also overexpressed by LCH cells compared with skin-resident LCs^25^. A unique characteristic of unconventional T cells is their ability to produce large amounts of T helper (Th) 1 (TNF/IFNγ), Th2 (IL-4), Th10 (IL-10), or Th17 (IL-17) cytokines^29–31^ (also see review by Godfrey *et al*.^32^) depending on the setting. Consequently, unconventional T cells are a logical target of investigation in an inflammatory setting such as LCH. We therefore characterized the frequency and function of unconventional T cell lineages in blood and tissue lesions from patients with LCH and healthy individuals. We hypothesize that the immune dysregulation accompanying LCH progression is associated with abnormalities in unconventional T cells and that one or more of these lineages may represent a viable target for new treatments.

## Results

### Clinical details of patients with LCH

Our cohort included 11 male and 5 female patients with LCH aged from 22 months to 68 years (Table 1). The most commonly affected tissues were bone, skin and lung. With the exception of one patient, donors with active disease (AD) had no known prior treatment with steroids or chemotherapy at the time of specimen collection, but the majority of those with non-active disease (NAD) had received treatments (Table 1).

**Table 1:** Clinical details of study participants.

Patient Code	Specimen description	Sex	Age at diagnosis	Tissues affected	Treatment prior to specimen	Age at specimen	Disease status at specimen	Sequelae, other comments
1	Bone lesion	M	68	Bone	Previous irradiation at site of and excision of hip lesion	68	Active (AD)	Diabetes insipidus from age 55
1	Matched blood							
2	Pulmonary lesion	M	40	Lung	Nil	40	AD	Mild pulmonary fibrosis, smoker
2	Matched blood							
3	Involved lymph node	M	9	Bone, lymph node	Nil	9	AD	Skull defect
4	Blood	M	18 months	Skin	Nil	13	Non-active (NAD)	
5	Blood	M	53	Bone, lung, bone marrow	Vinblastine/prednisolone	64	NAD	Mild pulmonary fibrosis
6	Blood	M	39	Lung	Vinblastine/prednisolone	52	NAD	
7	Blood	F	60	Skin	Methotrexate/prednisolone	64	NAD	Leg scarring
8	Blood	F	36	Bone	Lesion excision	37	NAD	
9	Blood	F	25	Bone	Vinblastine/prednisolone	42	NAD	Ataxia at time of specimen
10	Blood	M	41	Skin	Nil	41	AD	
11	Blood	F	39	Bone, lung	Nil	39	AD	
12	Blood	F	34	Lung	Biopsy and vinblastine/prednisolone	54	NAD	Long term pulmonary fibrosis
13	Bone lesion	M	2	Bone	Nil	2	AD	Mutation in BRAFV600, CNS-risk lesion
13	Matched blood							
14	Blood	M	8 months	Skin, lymph node, liver	Nil	22 months	AD	No detectable mutation in BRAFV600
15	Blood	M	10	Bone, skin	Cytarabine, prednisolone and vinblastine (ceased 6 months prior to specimen)	12	AD	Diabetes insipidus, mutation in BRAFV600
16	Blood	M	67	Bone, skin	Nil	67	NAD	

### Circulatory MAIT cell proportion is decreased in patients with LCH

**Figure 1:**
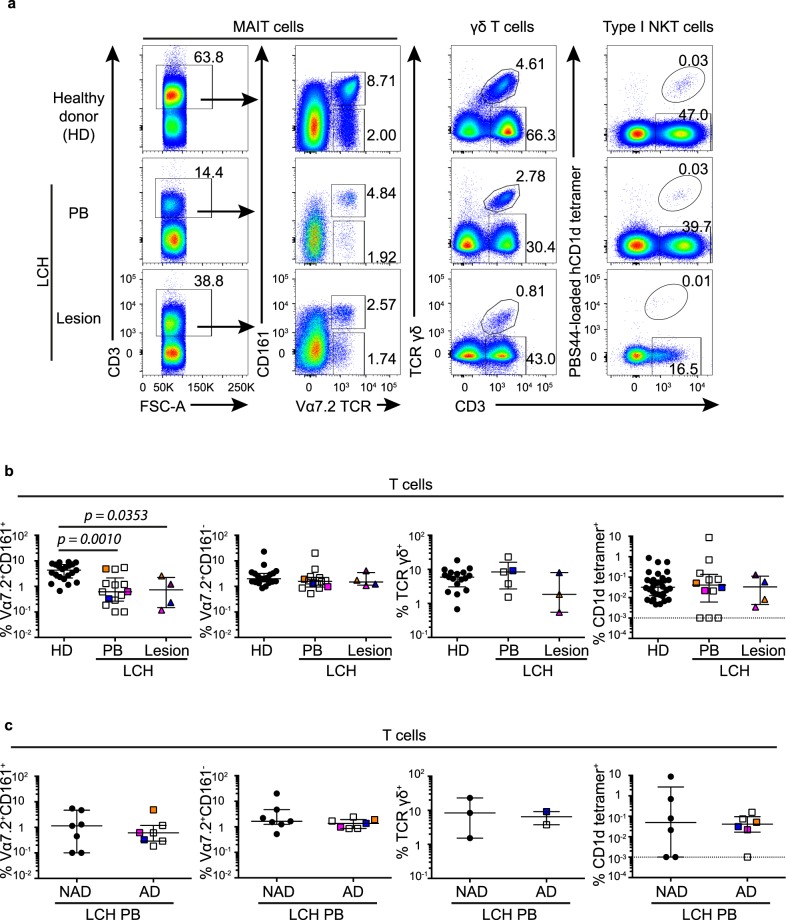
Circulatory and lesional unconventional T cell proportions in LCH. (**a**) Gating strategy for identifying MAIT cells (1^st^ and 2^nd^ columns), γδ T cells (3^rd^ column) and type I NKT cells (4^th^ column) from circulating lymphocytes in healthy donors (1^st^ row) and patients with LCH (2^nd^ row), and from lesional lymphocytes in patients with LCH (3^rd^ row). (**b**) Proportions of MAIT cells and Vα7.2 TCR^+^CD161^−^ T cells, γδ T cells and type I NKT cells in total T cells. (**c**) Proportions of MAIT cells, Vα7.2 TCR^+^CD161^−^ T cells, γδ T cells and type I NKT cells in circulating T cells from patients with LCH (LCH-PB) stratified by active disease (AD) and non-active disease (NAD). Kruskal-Wallis tests with Dunn’s multiple comparisons were conducted for (**b**) and Mann Whitney tests for (**c**), error bars indicate median + interquartile range, 10^−3^ on logarithmic scale indicates ‘undetectable’.

Unconventional T cells were stringently identified according to the following criteria: MAIT cells were classified as CD3^+^Vα7.2 TCR^+^CD161^+^ lymphocytes, γδ T cells as CD3^+^γδ TCR^+^ and NKT cells as CD3^+^PBS44-loaded CD1d-tetramer^+^ lymphocytes. Specimens analyzed were from the blood of healthy donors and patients with LCH, and from lesions from patients with LCH (Fig. 1a). The frequency of MAIT cells expressed as a proportion of total T cells was significantly lower in blood (*p* = *0*.*0010*) and lesions (*p* = *0*.*0353*) from patients with LCH compared to healthy donors (Fig. 1b). The measure of MAIT cell frequency can sometimes be confounded by downregulation of CD161, however the proportion of Vα7.2 TCR^+^CD161^−^ T cells did not increase in blood and lesions from LCH donors (Fig. 1b). No substantial difference was seen in the proportions of γδ T cells or NKT cells across the groups (Fig. 1b). Interestingly, there was greater variability in the proportion of circulatory NKT cells in patients with LCH (*range* = *0*.*000% to 8*.*68%*) compared to healthy donors (*range* = *0*.*005% to 0*.*871%*) (Fig. 1b). Blood samples from patients with LCH were further stratified into AD and NAD, however there were no differences in the proportions of circulatory MAIT cells, γδ T cells or NKT cells (Fig. 1c). Thus, while no discernible differences were observed in unconventional T cells between samples from AD and NAD donors, MAIT cells were reduced in samples from LCH donors compared to healthy blood donors.

### Comparing MAIT cell identification methods and proportions across age groups

**Figure 2:**
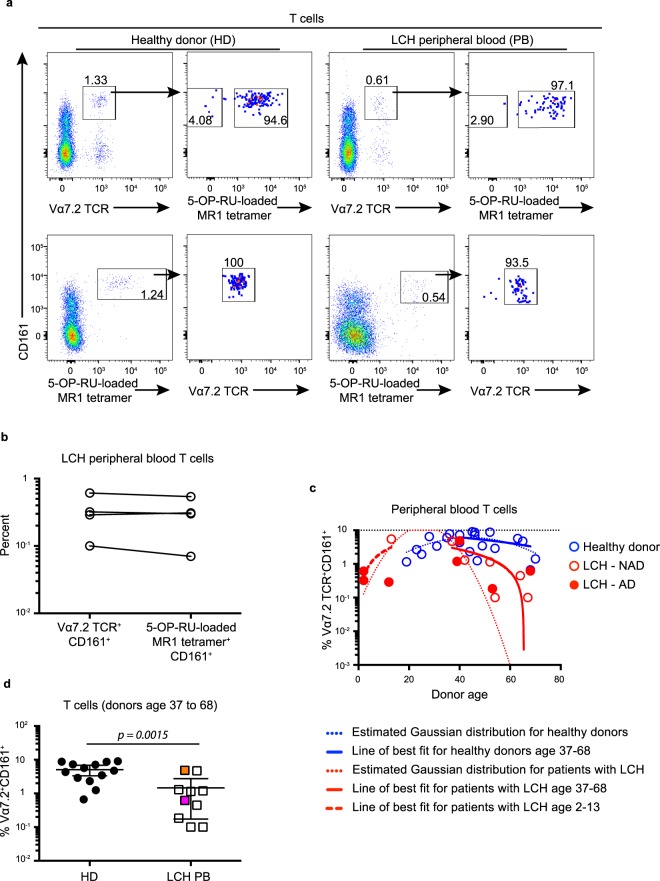
Comparing MAIT cell proportions across different identification methods and age groups. (**a**) Gating strategy for identifying 5-OP-RU-loaded MR1 tetramer^+^ cells within the Vα7.2 TCR^+^CD161^+^ T cell population (top row) and Vα7.2 TCR^+^ cells within the 5-OP-RU-loaded MR1 tetramer^+^CD161^+^ T cell population (bottom row) in peripheral blood from healthy donors (left) and patients with LCH (right). Data are representative of at least 3 individual donors per group. (**b**) Matched proportions of Vα7.2 TCR^+^CD161^+^ T cells and 5-OP-RU-loaded MR1 tetramer^+^CD161^+^ T cells in total T cells from the peripheral blood of patients with LCH. (**c**) Proportions of MAIT cells in peripheral blood T cells from healthy donors (blue circles) and patients with active (AD) LCH (closed red circles) and non-active (NAD) LCH (open red circles) with imposed Gaussian distributions for each data set (dotted lines) and a line of best fit for healthy donors (solid blue line) and LCH patients (solid red line) aged 37–68, and for LCH patients aged 2–13 (red dashed line). (**d**) Proportions of MAIT cells in total T cells from the peripheral blood from healthy donors aged 37 to 68 and patients with LCH aged 37 to 68. An unpaired t Test with Welch’s correction was conducted for (**d**), error bars indicate mean + 95% confidence interval.

We identified MAIT cells as CD3^+^Vα7.2 TCR^+^CD161^+^ lymphocytes, however a recently developed alternative is the use of 5-OP-RU-loaded MR1 tetramer^33^. We conducted an analysis to confirm that both approaches identify the same cells. Indeed, we found that Vα7.2 TCR^+^CD161^+^ MAIT cells were almost exclusively 5-OP-RU-loaded MR1 tetramer^+^ and 5-OP-RU-loaded MR1 tetramer^+^ MAIT cells were almost all Vα7.2 TCR^+^CD161^+^ (Fig. 2a), and importantly, that both approaches yielded similar data in analysis of LCH samples (Fig. 2b).

We also noted that MAIT cell frequency can fall with age in healthy individuals^34,35^, so it was important to establish if the abnormalities we observed in LCH patients may have been influenced by age. MAIT cell proportions were plotted against age and a line of best fit was determined for healthy donors and for patients with LCH between the ages 37 to 68 (Fig. 2c). For healthy donors, a Spearman’s non-parametric correlation test (95% confidence interval) determined that MAIT cell proportion was not significantly (*p* = *0*.*31*) correlated to age (*r* = *−0*.*3*), while in patients with LCH, MAIT cell proportion was significantly (*p* = *0*.*01*) negatively correlated to age (*r* = *−0*.*78*) (Fig. 2c). Although there was no significant correlation for healthy donors, we do not exclude the possibility that an association may have been identified with an increased cohort and indeed, our data did suggest a slight trend. Importantly, however, there remained a significant (*p* = *0*.*0015*) decrease in the proportion of MAIT cells in total T cells when we compared the peripheral blood from patients with LCH age 37 to 68 compared to healthy donors age 37 to 68 (Fig. 2d). Therefore age related changes in MAIT cell frequency do not account for the abnormalities we have observed in patients with LCH.

### CD4^+^Vα7.2 TCR^+^CD161^+^ T cells are increased in patients with LCH

**Figure 3:**
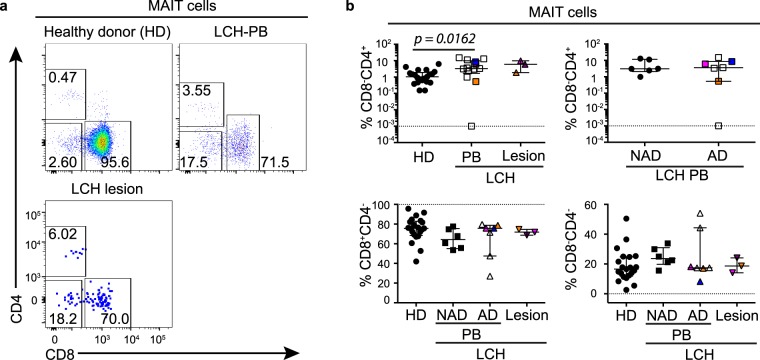
MAIT cell subsets in LCH. (**a**) Gating strategy for identifying CD8 and CD4 cells in MAIT cells from the peripheral blood from healthy donors (top left) and LCH patients (PB) (top right), and in lesional MAIT cells from patients with LCH (bottom left). (**b**) Proportions of CD4^+^, CD8^+^, and CD8^−^CD4^−^ cells in MAIT cells from the peripheral blood from healthy donors (HD), patients with non-active LCH (PB-NAD) and active LCH (PB-AD) and in lesional MAIT cells from patients with active LCH. Kruskal-Wallis tests with Dunn’s multiple comparisons were conducted for (**b**) except where only two groups were compared (Mann Whitney test), error bars indicate median + interquartile range, 10^−3^ on logarithmic scale indicates ‘undetectable’.

Given there are functionally distinct subsets of unconventional T cells, we next tested the relative frequency of several MAIT, NKT and γδ T cell subpopulations. MAIT cells were primarily identified as Vα7.2 TCR^+^CD161^+^ T cells and we examined CD8 and CD4 subsets (Fig. 3a) and found that patients with LCH had an increased proportion of blood CD4^+^Vα7.2 TCR^+^CD161^+^ T cells, and a similar trend was observed in lesions, although this was not significant (Fig. 3b). No differences were observed upon further stratification of LCH blood samples into AD and NAD (Fig. 3b). Although CD4^+^Vα7.2 TCR^+^CD161^+^ T cells are not exclusively 5-OP-RU-MR1 tetramer binding^35^, Eomes, PLZF and CCR4 expression is comparable in these populations irrespective of the gating method, thus CD4^+^Vα7.2 TCR^+^CD161^+^ T cells are phenotypically similar to CD4^+^ 5-OP-RU antigen specific MAIT cells^36^.

### Unusual γδ T cell and type I NKT cell phenotype in patients with LCH

**Figure 4:**
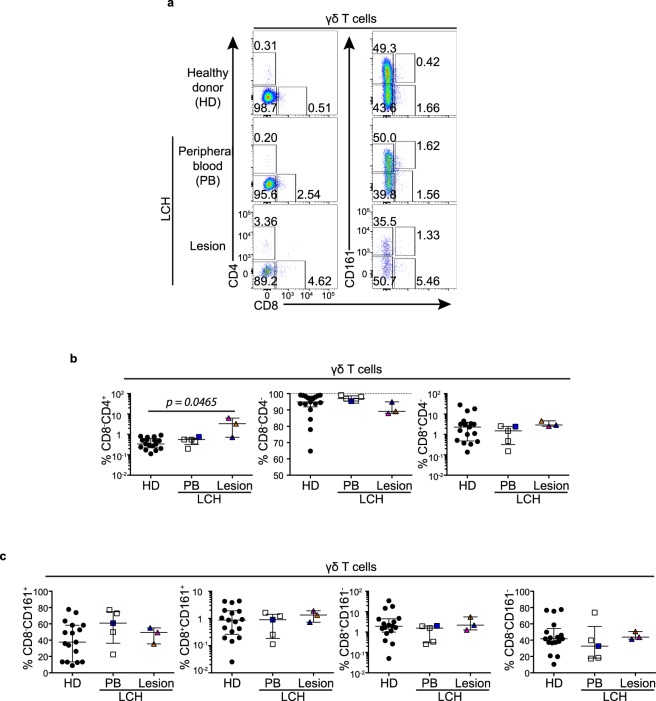
γδ T cell subsets in LCH. (**a**) Gating strategy for identifying γδ T cell subsets. (**b**) Proportions of CD4^+^CD8^−^, CD8^−^CD4^−^ and CD8^+^CD4^−^ cells in γδ T cells from the peripheral blood from healthy donors (HD) and patients with LCH (PB) and in lesional γδ T cells from patients with active LCH. (**c**) Proportions of CD8^−^CD161^+^, CD8^+^CD161^+^, CD8^+^CD161^−^ and CD8^−^CD161^−^ cells in γδ T cells from the peripheral blood from healthy donors (HD) and patients with LCH (PB) and in lesional γδ T cells from patients with active LCH. Kruskal-Wallis tests with Dunn’s multiple comparisons were conducted for (**b**,**c**), error bars indicate median + interquartile range.

Typically γδ T cells are CD8^−^CD4^−^, however there are known transcriptional differences between CD161^+^ and CD161^−^ γδ T cells^37^. Therefore we compared the proportions of CD8^−^CD4^−^, CD8^+^(CD4^−^) and CD4^+^(CD8^−^) γδ T cells and CD8^−^CD161^−^, CD8^+^CD161^−^ CD8^−^CD161^+^ CD8^+^CD161^+^ γδ T cell subsets in patients with LCH (Fig. 4a). We found a significant (*p* = *0*.*0465*) increase in the proportion of CD4^+^(CD8^−^) γδ T cells from LCH lesions compared with healthy donors (Fig. 4b) but no discernible differences were detected in the CD161 subsets between the healthy donor and LCH patient groups (Fig. 4c).

**Figure 5:**
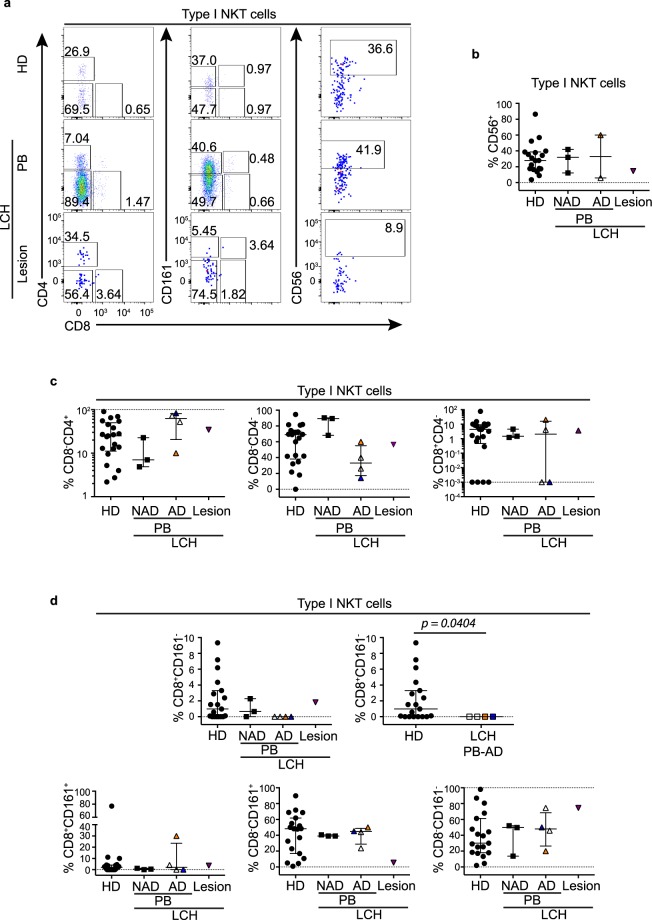
NKT cell subsets in LCH. (**a**) Gating strategy for identifying type I NKT cell subsets in peripheral blood from healthy donors (HD) and patients with LCH (PB), and in lesions from patients with LCH. (**b**) Proportions of CD56^+^ cells in type I NKT cells. (**c**) Proportions of CD4^+^CD8^−^, CD8^−^CD4^−^ and CD8^+^CD4^−^ cells in type I NKT cells. (**d**) Proportions of CD8^+^CD161^−^, CD8^+^CD161^+^, CD8^−^CD161^+^ and CD8^−^CD161^−^ cells in type I NKT cells. Kruskal-Wallis tests with Dunn’s multiple comparisons were conducted for (**b**–**d**) (LCH lesion group excluded due to sample size) except where only two groups were compared (Mann Whitney test), error bars indicate median + interquartile range, 10^−3^ on logarithmic scale indicates ‘undetectable’.

For NKT cells we investigated CD8 and CD4 subsets and CD56 expression (Fig. 5a) and found that while there were similar proportions of circulatory CD56^+^ NKT cells (Fig. 5b) and CD8^+^ NKT cells (Fig. 5c) between patients with LCH and healthy donors there were trends toward a higher proportion of CD4^+^ and a lower proportion of CD8^−^CD4^−^ NKT cells in blood from patients with AD compared to NAD (Fig. 5c). Interestingly we observed reduced circulatory CD8^+^CD161^−^ NKT cells in patients with AD compared to healthy donors (Fig. 5d).

### Activation status of unconventional T cells in patients with LCH

**Figure 6:**
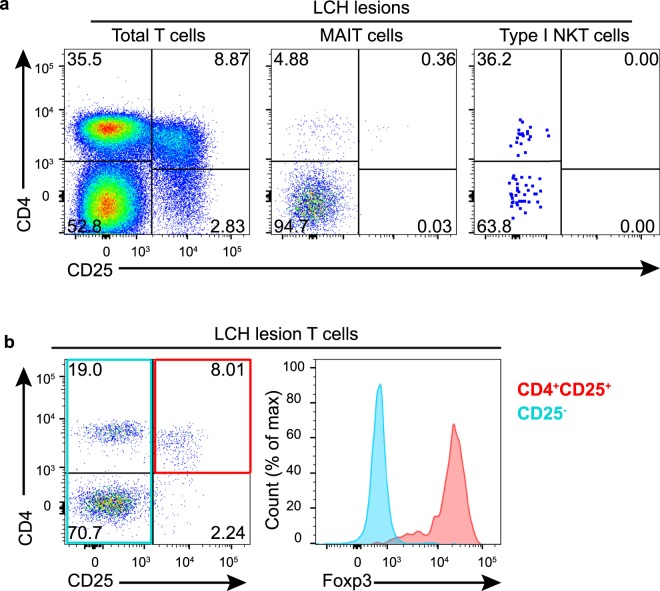
Activation status of T cells in patients with LCH. (**a**) Representative plots demonstrate that most CD25^+^ T cells in LCH lesions are CD4^+^ (left) and there is little to no CD25 expression by MAIT cells (center) or type I NKT cells (right). (**b**) Representative plots demonstrate that most CD25^+^ T cells in lesions are CD4^+^Foxp3^+^. Data are representative of at least 4 individual donors in (**a**) and 3 individual donors in (**b**).

LCH lesions are thought to harbor activated T cells^5,13,16^ and we were therefore assessed CD25 expression in LCH donors. We unexpectedly did not identify any considerable CD25 expression by either MAIT cells or NKT cells in LCH lesions (Fig. 6a) and most CD25^+^ T cells within LCH lesions were CD4^+^Foxp3^+^ Tregs (Fig. 6b).

### Expression of *MR1* by LCH cells

While γδ T cells can be stimulated by antigen independently of MHC or MHC-like molecules, CD1d and MR1 are necessary for antigenic stimulation of NKT cells and MAIT cells respectively. It is already known that *CD1D* is expressed by LCH cells^25^, but MR1 has not been directly studied in the context of LCH. *MR1* is ubiquitously expressed but it was possible that a defect could impact MAIT cells, so we tested for the expression of *MR1* by purified LCH (CD1a^+^) cells using qPCR. We found that *MR1* was expressed by LCH cells (delta Ct from reference gene *RPLP0* values: −2.24 and −7.97). These results suggest that LCH cells can express MR1 and could potentially present MR1-restricted antigens to MAIT cells in lesions. Importantly, MAIT cells can also be activated independently of antigen by IL-12 and IL-18, which is noteworthy because there are elevated levels of IL-18 in the peripheral blood from patients with LCH^38^.

### Unconventional T cells from patients with LCH can produce Th1 cytokines

**Figure 7:**
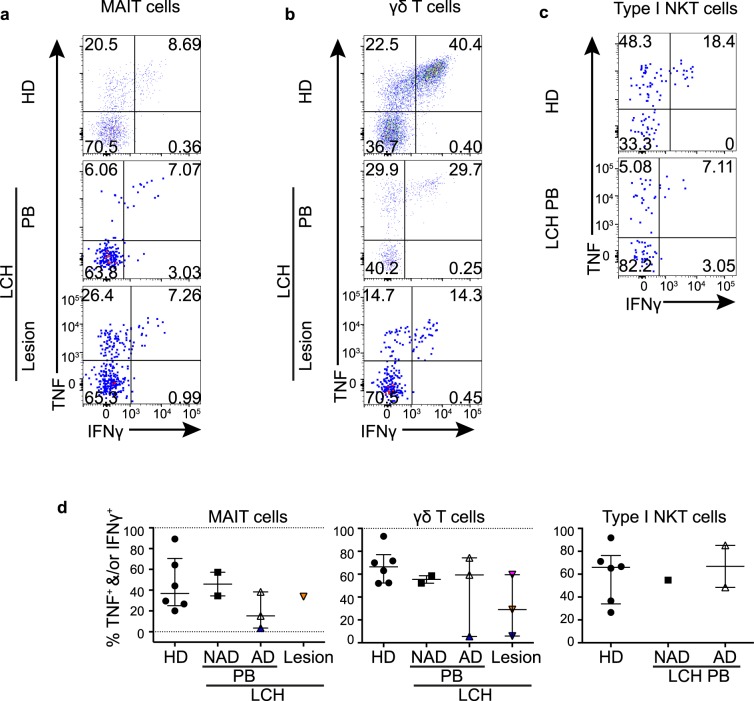
Th1 cytokine production by unconventional T cells. Gating strategy for post stimulation identification of TNF and IFNγ producing MAIT cells (**a**), γδ T cells (**b**) and type I NKT cells (**c**) from the peripheral blood from healthy donors (HD) and patients with LCH (PB), and from lesional T cells from patients with active LCH. (**d**) Proportions of TNF^+^ and/or IFNγ^+^ T cells in MAIT cells, γδ T cells and type I NKT cells from the peripheral blood from healthy donors (HD), patients with non-active LCH (PB-NAD) and active LCH (PB-AD) and in lesional T cells from patients with active LCH. Kruskal-Wallis tests with Dunn’s multiple comparisons were conducted for (**d**) (groups with only one value excluded), except where only two groups were compared (Mann Whitney test), error bars indicate median + interquartile range, 10^−3^ on logarithmic scale indicates ‘undetectable’.

Next, we investigated the functional capacity of unconventional T cells from LCH patients as it was important to establish if these cells could mount a normal cytokine response to stimulation. We stimulated T cells with PMA and ionomycin and examined TNF and IFNγ production by MAIT cells (Fig. 7a), γδ T cells (Fig. 7b) and NKT cells (Fig. 7c). MAIT cells, NKT cells and γδ T cells from patients with LCH were able to produce TNF and IFNγ, albeit in small proportions from one donor in particular (Fig. 7d). IL-4 and IL-17A cytokine production was negligible by all T cells in LCH and healthy donors (data not shown). Our data suggest that although decreased in proportion, the MAIT cells in patients with LCH are functionally competent. It is important to note, however that more subtle defects may become evident with different forms of activation.

### Relative frequency of circulatory MAIT cells during AD and NAD and the effects of treatment

**Figure 8:**
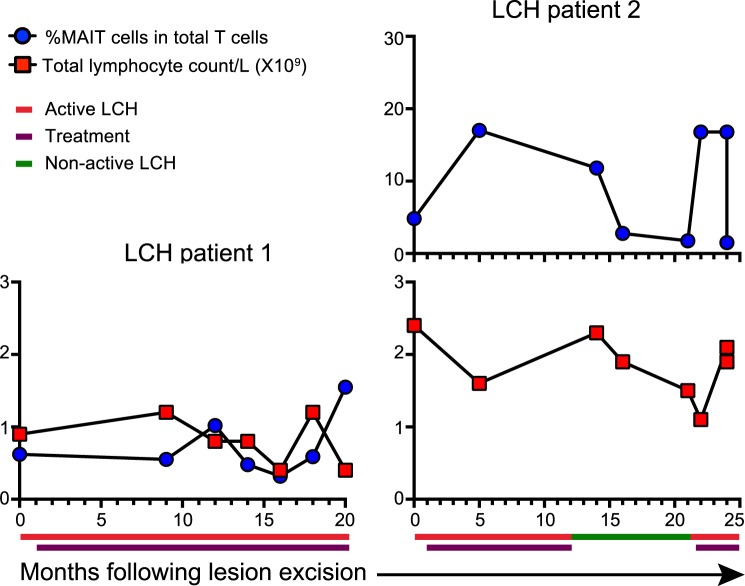
Relative frequency of circulatory MAIT cells over time. Graphs show proportions of MAIT cells in total T cells and the corresponding total lymphocyte counts over a period of months following lesion excision and during chemotherapy. Patient 1 received vinblastine or methotrexate and prednisolone for the duration of the period. Patient 2 received vinblastine and prednisolone.

Given our observation that MAIT cells are reduced in the circulation of patients with LCH, and their known immunoregulatory ability, we conducted a longitudinal analysis of MAIT cells at different stages of disease and treatment following lesion excision and during chemotherapy in two patients at a number of time points for a period of 20 to 25 months (Fig. 8). We observed minor changes in the MAIT cell proportion and total lymphocyte count of one patient with AD who was receiving vinblastine or methotrexate and prednisolone. More interestingly, the MAIT cell frequency of the other patient increased during treatment with vinblastine and prednisolone and decreased at cessation of treatment when the patient had NAD. Furthermore, this patient’s MAIT cell proportion increased when the disease reactivated and treatment recommenced. Whether the changes in MAIT cell proportion were disease specific or treatment specific was not established, but this is an indication that MAIT cell frequency could potentially be associated with LCH disease activity.

## Discussion

Unconventional T cells include several immunoregulatory lineages but have been difficult to study due to low numbers and a lack of reagents to enable stringent identification and functional analysis. Improved knowledge about these cells and our access to important reagents enabled us to characterize MAIT cells, NKT cells and γδ T cells and determine if there were defects in their frequency or cytokine production.

MAIT cells were reduced in proportion in the blood from patients with LCH and the lesional proportions were consistent with that seen in the blood from patients. Interestingly, the proportion of circulating MAIT cells was similar in patients with AD and NAD, which suggests that a MAIT cell deficiency may precede the onset of AD and could be a potential predisposing factor for LCH. Of note, longitudinal analysis of one patient revealed a substantial rise in MAIT cell frequency after treatment and a subsequent fall when treatment ceased and the disease was non-active. Fluctuations in MAIT cell proportion, which were remarkably at approximately 17% of total T cells for three separate time points, will be interesting to pursue in a cohort of LCH donors. Albeit in a single donor, the changes are consistent with our hypothesis that increasing MAIT cell frequency could be beneficial in the resolution of LCH. It is vital to emphasise that the result is a very preliminary observation. We did not observe an increase in γδ T cells reported by Alaibac and Chu^24^, although an important difference in our study was that we assayed lesions from various tissue sites, whereas the earlier study was limited to cutaneous lesions.

Unconventional T cells include several functionally distinct lineages and it was noteworthy that there were changes in subset proportions of γδ T cells and NKT cells as well as in the relative frequency of MAIT cells. γδ T cells do not usually express CD4, but we unexpectedly identified an increase in the proportion of lesional CD4^+^ γδ T cells. Determining the cause and functional significance of this is outside the scope of this study, but it was interesting that we also detected an increase in the proportion of CD4^+^Vα7.2 TCR^+^CD161^+^ T cells in patients with LCH. As with γδ T cells, the CD4^+^Vα7.2 TCR^+^CD161^+^ T cells are usually a minor subset, but their increase in LCH parallels a similar observation reported for patients with colorectal cancer^23^. It is important to note, however, that this increase in CD4^+^Vα7.2 TCR^+^CD161^+^ T cells may be due to the overall reduction in MAIT cells.

The frequency of circulating NKT cells in healthy donors usually ranges from 0.01 to 0.1% of overall T cells, but is rarely greater than 1%^39^. Surprisingly, NKT cell frequency in patients with LCH was more variable and even extended to a patient where NKT cells comprised almost 9% of T cells in blood. A previous case study of an individual with 5% NKT cells was considered exceptional^39^ and it is noteworthy that the patient in our study had NAD at the time of sample collection, which followed a spontaneous disease resolution without treatment. Because NKT cells are immunoregulatory in nature, it would be interesting to determine if the high NKT cell frequency was pre-existing or accompanied this remission, which may indicate a direct role for NKT cells. In striking contrast were several patients with LCH that had undetectable NKT cells. This too is unusual in healthy individuals and such a deficit could be expected to have a large impact on immune regulation and potentially the progression of LCH. The factors controlling NKT cell frequency in humans are poorly understood but once determined, it will be interesting to assess if they are defective in LCH patient groups.

We investigated whether the frequency abnormalities of unconventional T cells extended to their functional capacity, as this has previously been reported for many patient groups, including with cancer^23,40^. Each T cell lineage that we studied from patients with LCH retained the capability to produce Th1 cytokines after stimulation. High levels of IL-17A have been reported in the blood of patients with LCH^41^ and more controversially^25,42^ in lesions^43^. Our findings of negligible IL-17A production by T cells from patients with LCH indicates that T cells in LCH do not respond with rapid IL-17A release, although we cannot rule out that an *in vivo* stimulus could cause IL-17A production.

Having identified these immune abnormalities, it will be essential for future studies to determine their origin and whether they precede LCH, or are caused by it. An intriguing question is whether unconventional T cells are directly interacting with LCH cells and if so, whether this results in T cell activation, or affects the LCH cells. For example, in addition to cytokine release, activated MAIT cells express CD40L^44^, which can mature LCH cells *in vitro* and increase their allostimulatory capacity^45^. This implies that LCH cells could mature and present antigen more effectively in the presence of activated MAIT cells, which could promote inflammation and reduce Treg induction by immature myeloid cells, in turn resolving lesions.

We have shown for the first time that the frequency of unconventional T cells, especially MAIT cells, is abnormal in patients with LCH. MAIT cell frequencies appear similar in the periphery of patients with AD and NAD, although an increase in proportion was observed upon treatment in one patient that suggested MAIT cells could be associated with LCH onset and progression, although further studies are required to confirm this. Changes in unconventional T cell subset distributions and frequency in patients with LCH are consistent with the established role of unconventional T cells in immune dysregulation. It will be especially informative to determine the direct roles of these cells in LCH and whether the abnormalities we have identified are a cause or consequence of the disease. Our analysis strengthens earlier reports of immune dysfunction in LCH by showing the abnormalities extend beyond Foxp3^+^ Tregs to unconventional T cell lineages that could be equally important factors in LCH. Their distinctive functions and the development of specific agonists make unconventional cells promising candidates as biomarkers or targets of immune based therapies, but more detailed analysis is now required for translation to clinical applications.

## Materials and Methods

### Human blood and tissue

This research was approved by the Ballarat Health Services and Saint John of God Ballarat Hospital Human Research Ethics Committee (HREC) (HREC/15/BHSSJOG/5 and HREC/10/BHSSJOG/57) and Federation University Australia HREC (A08-100) and the methods were carried out in accordance with the approved guidelines. Patients, and/or parents of children with LCH where appropriate, provided written informed consent. Healthy donor peripheral blood mononuclear cells (PBMCs) were obtained from the Australian Red Cross Blood Service.

Healthy donor PBMCs were isolated with Histopaque-1077 (Sigma-Aldrich). PBMCS were isolated from the peripheral blood from patients with LCH using Histopaque-1077 or Lymphoprep (Axis-Shield PoC AS, Oslo, Norway), otherwise white blood cells were isolated using an in house red cell lysis buffer. Tissue was processed using MACS tumour dissociation kit for humans (Miltenyi Biotec) as per manufacturer’s instructions. Different methods were used to achieve a single cell suspension for lesions and for some LCH peripheral blood samples compared to healthy donors, therefore flow cytometry analysis was conducted relative to the CD3^+^ (T cell) population or sub-populations thereof. LCH lesions and matched peripheral blood samples are colour coded for tracking throughout the figures.

### Surface antibody staining

Cells were stained with viability dye 7-aminoactinomycin D (7-AAD; BD Pharmingen) and anti-human cell surface antibodies from the following list: CD1a-BV605 (SK9; BD Biosciences),CD3-Pacific Blue (HIT3a; BioLegend), CD3-BV605 (HIT3a; BD Horizon), CD3-BV650 (UCHT1; BD Horizon), CD4-PE-Cy7 (SK3; BD Pharmingen), CD4-BV650 or -BV711 (SK3; BD Horizon), CD8-BV510 (RPA-T8; BD Horizon), CD25-PE-Cy7 (M-A251; BD Pharmingen), CD56-BV786 (NCAM16.2; BD Horizon), CD127-BV421 (HIL-7R-M21; BD Horizon), CD161-APC or -PE/Dazzle 594 (HP-3G10; BioLegend), TCR Vα7.2-FITC (3C10; BioLegend), and TCRγ/δ-1-PE-Cy7 (11F2; BD Biosciences).

For identification of NKT cells, cells were stained with PBS44 loaded human CD1d tetramers. PBS44-CD1d monomers were kindly donated by Dale Godfrey (Dept. Microbiology and Immunology, Peter Doherty Institute, University of Melbourne, Parkville, Australia). In some instances, MAIT cells were identified using 5-(2-oxopropylideneamino)-6-D-ribitylaminouracil (5-OP-RU) loaded human MR1 tetramers. 5-OP-RU-MR1 monomers were a kind gift from the McCluskey laboratory (Dept. Microbiology and Immunology, Peter Doherty Institute, University of Melbourne, Parkville, Australia). CD1d and MR1 monomers were tetramerized by conjugation to PE streptavidin (BD Pharmingen).

### Foxp3 transcription factor staining

Following surface staining the eBioscience™ Foxp3/Transcription Factor Staining Buffer Set (ThermoFisher Scientific) was used as per manufacturer’s instructions.

### Flow cytometry and fluorescence activated cell sorting

Analyses were performed using either a BD FACS Aria II cell sorter or BD LSR Fortessa. All cell sorting was performed using a BD FACS Aria II cell sorter and data analyzed using FlowJo software (Treestar). All analyses excluded dead cells and doublets.

### RNA extraction, cDNA synthesis, transcriptome amplification and qPCR

Cells were purified by FACS (purity >90%), then the REPLI-g WTA Single Cell Kit (QIAGEN) for transcriptome amplification of small cell populations (<1000) was used to extract and reverse transcribe total RNA and amplify cDNA directly from cells, as per manufacturer’s instructions using a thermal cycler (Applied Biosystems Gene Amp® PCR System 2700). RT_2_ SYBR® Green qPCR Mastermix (QIAGEN), Nuclease-Free Water (QIAGEN) and RT_2_ qPCR Primer Assays for *MR1* (QIAGEN) and reference gene *RPLP0* (QIAGEN) were combined with cDNA as per manufacturer’s instructions. No-template control samples were included for each component, substituting cDNA volumes with nuclease free water. Aliquots of each component were added to 0.1 mL strip tubes (QIAGEN) in triplicate and capped then placed in the Rotor-Disk 72 Rotor (QIAGEN) and placed in the Rotor-Gene Q (QIAGEN) for qPCR cycling. Conditions for amplification, annealing, extension and melt curve analysis were as per QIAGEN RT_2_ qPCR primer assay instructions. Melt curve analysis was performed to confirm that specific product was amplified as indicated by the data sheet for each primer assay.

### Unconventional T cell stimulation assay

CD3^+^ T cells were sorted with a purity above 95%, except from two LCH peripheral blood samples, which were above 92%, and rested overnight at 37 °C in TexMACS Medium (Miltenyi Biotec) with 10% Fetal Bovine Serum (Sigma-Aldrich) and 1x Penicillin-Streptomycin (Sigma-Aldrich), then stimulated for 5 hours with PMA (10 ng/mL) and ionomycin (1 μg/mL) in the immediate presence of GolgiPlug (BD Biosciences).

### Fixation, permeabilization and intracellular cytokine staining

Following stimulation assays and surface staining, cells were fixed and permeabilized using BD Cytofix/Cytoperm kit (BD Biosciences) as per manufacturer’s instructions. Cells were then stained for intracellular cytokines from the following list: IL-4-APC (BD Pharmingen), TNFα-APC-Cy7 (MAb11; BioLegend), IFNγ-BV711 (B27; BD Horizon) IL-17A-BV786 (N49-653; BD Horizon).

### Statistics

Data were analyzed using GraphPad Prism (GraphPad Software). Three donor matched blood and LCH lesions were available for the initial characterization of MAIT cells and NKT cells and one for the characterization of γδ T cells and the PMA and ionomycin stimulation of unconventional T cells. D’Agostino and Pearson omnibus normality tests (alpha = 0.05), were used to select parametric or non-parametric analysis. Differences were considered statistically significant at an alpha level of 0.05.

## Data Availability

The datasets generated during and/or analyzed during the current study are available from the corresponding author on reasonable request.
